# Health Literacy Prediction Based on Information Literacy and Physical Literacy Components: A Cross‐Sectional Study

**DOI:** 10.1002/hsr2.72303

**Published:** 2026-04-27

**Authors:** Maryam Mohammadi, Sahar Mohammadnabizadeh

**Affiliations:** ^1^ Department of Health Education and Health Promotion, School of Health Mashhad University of Medical Sciences Mashhad Iran; ^2^ Social Determinants of Health Research Center Mashhad University of Medical Sciences Mashhad Iran

**Keywords:** health, health literacy, information literacy, literacy, physical literacy

## Abstract

**Background and Aims:**

Despite the growing recognition of the importance of physical literacy, information literacy, and health literacy, there is a lack of research on their relationships among university staff. This study aimed to investigate the predictive relationship between health literacy and the components of information literacy and physical literacy among staff at university.

**Methods:**

Using a cross‐sectional design and convenience sampling, the study collected information among 250 administrative university staff via demographic, health literacy, information literacy, and physical literacy instruments. To identify predictors of health literacy, a multiple linear regression model was fitted with health literacy as the dependent variable. Statistical analysis was conducted with SPSS.

**Results:**

The mean scores for health literacy, physical literacy, and information literacy were 3.98, 3.31, and 3.08, respectively. The results showed that health literacy is significantly and positively associated with information literacy (β = 0.84, *p* = 0.001) and education (β = 0.14, *p* = 0.02). The findings indicated that information literacy was a strong predictor of health literacy, with no significant relationships found between physical literacy, age, marital status, and gender, and health literacy.

**Conclusion:**

Information literacy was a robust predictor of health literacy, independent of physical literacy, age, marital status, or gender. Education also contributed to health literacy, albeit to a modest extent. Interventions aiming to boost health literacy should prioritize strengthening information literacy skills, with considerations for educational background to optimize impact.

## 1 Introduction

In today's information‐rich environment, knowledge and literacy across multiple domains shape individuals' ability to access, interpret, and apply information to promote health and well‐being. Literacy in physical activity, information use, and health is increasingly essential as social, economic, and technological changes accelerate the need for competent decision‐making and healthy lifestyles [1].

Health literacy is a critical determinant of health, with a well‐documented social gradient: individuals from lower socioeconomic backgrounds often exhibit lower health literacy, which is associated with poorer health outcomes [2], higher mortality, and greater healthcare costs, particularly among older adults who are especially vulnerable [3]. Health literacy encompasses accessing, understanding, appraising, and applying health information, and deficits in these skills can have wide‐ranging personal and system‐level consequences [4, 5]. The consequences of low health literacy are far‐reaching, including poorer mental and physical health status, increased mortality, and higher healthcare costs [6]. These concerns motivate targeted interventions to raise health literacy, with emphasis on vulnerable populations [2, 7].

Physical literacy provides the foundation for engaging in physical activity and contributing to favorable health, social, and mental health outcomes [8]. It comprises physical competence, confidence, motivation, understanding, and knowledge necessary to participate in and sustain physical activity [9]. Strengthening physical literacy may yield improvements in cardiovascular and metabolic health and health‐related quality of life, and it can also influence educational outcomes and public health systems [10]. The relationship between physical literacy and health literacy has garnered increasing scholarly attention, with evidence indicating that health literacy is associated with nutritional behaviors, physical activity, and health‐related quality of life [11, 12, 13].

Information literacy, defined as the ability to locate, evaluate, and responsibly use information from diverse sources, has become indispensable in the information age [1]. High information literacy supports better health literacy, as individuals who can critically appraise information are better prepared to make informed health decisions [14, 15]. A constructive integration of information literacy with physical literacy offers a practical framework for promoting health literacy: information literacy enables critical evaluation of health information, while physical literacy grounds individuals in health‐promoting behaviors [16]. Accordingly, comprehensive educational programs that strengthen both information literacy and physical literacy can lay a foundation for advancing health literacy within health and educational systems [17].

### 1.1 Aims of the Study

Despite the growing recognition of the importance of physical literacy, information literacy, and health literacy, there is a lack of research on their relationships among university staff. This study aimed to investigate the predictive relationship between health literacy and the components of information literacy and physical literacy among staff at the university.

## 2 Materials and Methods

### 2.1 Study Design and Sampling

The current investigation employed a cross‐sectional survey design to study the predictive relationship between health literacy and the components of information literacy and physical literacy among administrative university staff, 2025. Due to a lack of access to an employee roster and relying on individuals' willingness to participate, we employed a convenience sampling approach among administrative staff at [Name of Institution], a medical sciences university in Iran.

Inclusion criteria were as follows [1]: no history of specified illnesses [2], willingness to participate, and [3] non‐participation in related training within the study period. Conversely, participants who submitted incomplete questionnaires were excluded from the study. Data collection was conducted through self‐reporting, where participants completed the questionnaire in a face‐to‐face format.

The sample size was calculated using Cochran's formula with a 5% margin of error and 95% confidence, resulting in a required sample of 227 individuals. To accommodate an anticipated 10% attrition, the target sample was set at 250 individuals:

$$n=\frac{\frac{z^{2}pq}{d^{2}}}{1+\frac{1}{N}\left[\frac{z^{2}pq}{d^{2}}-1\right]}$$

### 2.2 Ethics Approval and Consent to Participate

The study was approved by the Mashhad University of Medical Sciences ethics board (Number: IR. MUMS. FHMPM. REC.1404.111). In this study, the principles of the Declaration of Helsinki have been followed. A written consent was obtained from all participants after explaining the aim of the study.

### 2.3 Data Collection Tools

Demographic questionnaire: The demographic questionnaire was designed to collect a range of demographic data from participants, including gender, age, marital status, and education level.

**Health literacy questionnaire:** The health literacy of participants was assessed using the Iranian adult's health literacy questionnaire, which was developed and validated by Montazeri et al. [18]. The questionnaire exhibited strong psychometric properties, evidenced by a Cronbach's alpha coefficient of *α* = 0.90, indicating high internal consistency and reliability. The questionnaire consists of 33 questions that evaluate various dimensions of health literacy, including, **Availability of health information:** This dimension assesses the extent to which individuals have access to health information and can identify reliable sources of health information; **Reading comprehension:** This dimension evaluates the ability of individuals to read and understand health‐related texts; **Understanding:** This dimension assesses the ability of individuals to understand and interpret health information, including the ability to recognize the meaning of words, phrases, and sentences, and to understand the relationships between different pieces of information; **Evaluation:** This dimension assesses the capacity to critically appraise health information; **Behavioral intention:** This dimension measures the extent to which individuals intend to adopt healthy behaviors. Responses to the questionnaire were scored on a five‐point Likert scale, from “very little” to “very much”.

**Information literacy questionnaire:** The information literacy of participants was assessed using the Yazdani information literacy questionnaire, which has been previously validated [19]. To establish the questionnaire's reliability, a pilot study was conducted with 30 members of the study population, yielding a Cronbach's alpha of 0.80, which indicates good internal consistency. The questionnaire consists of 5 dimensions and 30 items, which are designed to evaluate various aspects of information literacy, including, **Information needs (5 items):** This dimension assesses the ability to recognize and articulate information needs, and to identify the types of information required to address those needs; **Information location (10 items):** This dimension evaluates the ability to locate and access relevant information from various sources; **Information evaluation (2 items):** This dimension assesses the ability to critically evaluate the credibility and relevance of information, and to recognize biases and limitations; **Information organization (7 items):** This dimension evaluates the ability to organize and structure information, and to create meaningful relationships between different pieces of information; **Information exchange (6 items):** This dimension assesses the ability to communicate and share information effectively with others, and to use information to inform decision‐making and problem‐solving. Responses to the questionnaire were measured using a 5‐point Likert scale, with endpoints 1 = strongly disagree and 5 = strongly agree.

Physical literacy questionnaire: The physical literacy of participants was assessed using Choi et al.'s physical literacy questionnaire [20], which has been adapted and validated for use in Iran by Soltani et al. [21]. To establish the reliability of the questionnaire, a pilot study was conducted with 30 members of the study population, yielding a Cronbach's alpha of 0.90, which indicates excellent internal consistency. The questionnaire consists of three domains, which are designed to evaluate various aspects of physical literacy, including, **Self‐efficacy and confidence (3 items):** This domain assesses the individual's confidence in their ability to engage in physical activities and their perceived self‐efficacy in achieving physical goals; **Self‐expression (self‐assertion) (3 items):** This domain evaluates the individual's ability to express themselves through physical activities and their willingness to take risks and try new things; **Physical activity knowledge (3 items):** This domain assesses the individual's knowledge and understanding of physical activities, including their awareness of the benefits and risks associated with different types of physical activity. he responses to the questionnaire were measured using a 5‐point Likert scale, with endpoints at 1 = strongly disagree and 5 = strongly agree.

### 2.4 Data Analysis

Statistical analyses were conducted using SPSS Statistics for Windows, version 22. Descriptive statistics (frequencies, percentages, means, and standard deviations) were used to describe the sample for all study variables. To identify predictors of health literacy, a multiple linear regression model was fitted with health literacy as the dependent variable. Independent variables entered in the model were information literacy, physical literacy, gender, age, education level, and marital status. Residuals from the final multiple linear regression model were examined for normality through visual inspection of Q–Q plots and, where appropriate, the Shapiro–Wilk test. Results indicated no substantial deviation from normality. Scatterplots of each predictor versus the predicted health literacy values and residuals versus fitted values were examined to assess linearity. No major nonlinearity was detected. Hypotheses were tested at an alpha level of 0.05. All tests were two‐tailed. Reported statistics included standardized regression coefficients (*β*), *t*‐values, and *p*‐values, 95% confidence interval, along with model fit information (*R*²).

## 3 Result

The demographic characteristics of the research participants are presented in Table 1. In terms of educational attainment, the majority of participants (54%) held a Bachelor's degree, followed by those with a Master's degree (32.4%), a PhD (7.6%), and an Associate degree (4%). The sample was predominantly female, with 61.2% of participants identifying as female, compared to 38.8% who identified as male. The majority of participants (86%) were married, while 14% were single. The mean age of the participants was 41.90 ± 4.15 years.

**Table 1:** Demographic characteristics of the participants.

Age	Mean	Standard Deviation
Year	41.9	4.15

	Number	Percentage
Gender		
Male	97	38.8
Female	153	61.2
Education		
Associate degree	10	4
Bachelor science	140	54
Master science	81	32.4
PhD degree	19	7.6
Marital status		
Married	215	86
Single	35	14

The results presented in Table 2 show the mean and standard deviation values for the variables of health literacy, physical literacy, and information literacy. The mean scores for these variables were 3.98, 3.31, and 3.08, respectively. These results suggest that the participants in the study had relatively high levels of health literacy, with a mean score of 3.98 out of a possible 5.

**Table 2:** Mean and standard deviation of variables.

Variables	Mean	Standard deviation	Minimum	Maximum
Health literacy	3.98	0.99	1	5
Physical literacy	3.31	1.32	1	5
Information literacy	3.08	1.33	1	5

The regression analysis revealed several significant predictors of health literacy, as presented in Table 3. The results showed that higher levels of information literacy (*β* = 0.84, *p* < 0.001) and education (*β* = 0.14, *p* = 0.02) were significantly and positively associated with health literacy. This suggests that individuals with greater information literacy and higher levels of education tend to have better health literacy. Moreover, the results indicate that information literacy is a strong predictor of health literacy, with a standardized beta coefficient of 0.84. Additionally, the results found no significant associations between physical literacy, age, marital status, and gender, with health literacy.

**Table 3:** Rates of analyses regression.

Independent variable	*ß* standard	*t*	*P*	95% confidence interval		*R* ^2^	Dependent variable
				Lower bound	Upper bound		
Physical literacy	0.11	1.77	0.08	−0.01	0.17		
Information literacy	0.84	10.99	< 0.001	0.52	0.74		
Age	0.01	0.41	0.69	−0.01	0.02	0.67	Health literacy
Marital status	0.03	0.76	0.45	−0.13	0.29		
Education	0.14	−2.40	0.02	−0.38	−0.04		
Gender	0.07	1.70	0.07	−0.01	0.28		

## 4 Discussion

The present study found no significant relationships between gender and health literacy, which is at odds with previous global studies that have reported differences in health literacy between males and females. For example, a research on college students in Egypt reported higher health‐literacy levels among females compared to males, attributed to more frequent health‐information seeking by females [22]. Likewise, an investigation of adults in Korea found that females exhibited higher health literacy than males in understanding medical information [23]. This null finding contrasts with some international studies that report higher health literacy among females, possibly due to greater health‐information seeking or differential health‐information processing. Differences in population (staff vs. students or general adults), cultural context, sampling, measurement instruments, and analytic strategies (e.g., power, interactions, domain‐specific outcomes) likely contribute to these discrepancies. The absence of a gender effect in our study should not be taken as evidence that gender never influences health literacy; rather, any potential effect may be contingent on context or moderated by factors such as education, age, or digital access. Future work should test these moderators and, where feasible, examine domain‐specific health‐literacy components using harmonized measures across diverse populations.

The investigation found no significant relationship between physical literacy and health literacy, indicating that these two concepts are distinct and not strongly related. This finding aligns with previous studies that have reported a lack of association between physical literacy and health literacy. For example, a study by Tremblay et al found that physical literacy was not significantly related to health literacy in a sample of Canadian adults [8]. In contrast, several studies have demonstrated a significant association between physical literacy and health literacy. For example, Cairney et al. found that physical literacy served as a significant predictor of health literacy among Canadian children [24]. The divergent findings across studies may reflect measurement variance: physical literacy and health literacy are multifaceted constructs captured by instruments with different domain emphases, which can attenuate or inflate observed associations. Additionally, sample characteristics may contribute to inconsistency; for example, university staff might differ in physical literacy, health literacy, or related behaviors compared with broader or younger populations. Future research should (a) employ harmonized, theory‐driven measures across diverse samples, (b) test domain‐specific subcomponents of physical literacy (e.g., knowledge, motor competence, motivation) for differential associations with health literacy, and (c) consider developmental stage and contextual moderators that might illuminate when physical literacy relates to health literacy.

The present study found a significant and positive association between health literacy and information literacy, consistent with a substantial body of prior work. Information literacy—enabling individuals to locate, assess, and apply health information—emerges as a key facilitator of health literacy, contributing to more informed health decisions, better self‐management, and engagement in health‐promoting behaviors [25]. The association between information literacy and health literacy has been investigated in numerous studies, which report a positive and substantive correlation between these constructs [25, 26]. For example, a study by Moghaddaszadeh reported a significant positive association between staff health literacy skills and information literacy levels [16]. In a related study, Mahmoudi and Taheri identified a significant positive association between information literacy and health literacy among students [27]. Mechanistically, information literacy skills likely enhance both the access to and the critical appraisal of health information, potentially bolstering self‐efficacy in information Problem‐solving and decision‐making [28]. While the precise causal pathways require longitudinal or experimental evidence, our findings reinforce the rationale for integrating robust information‐literacy training into educational programs. Such training may strengthen information‐seeking, evaluation, and application skills, thereby improving health literacy and, in turn, health‐related outcomes [28]. The study's findings emphasize the importance of providing effective information literacy training programs to increase students' self‐efficacy and health literacy [16]. Such programs enable individuals to access, appraise, and apply health information, which promotes healthful behaviors and effective self‐management of health conditions. Future research should test mediation by self‐efficacy and examine whether domain‐specific information literacy components differentially predict health literacy in various populations.

The study found significant relationships between education and health literacy. The relationship between education and health literacy is complex and multifaceted. Education contributes to health literacy by imparting skills and knowledge for making informed health decisions and navigating the healthcare system. Higher educational attainment likely enhances health literacy through multiple pathways, including improved critical thinking, information appraisal, and broader access to credible information sources, which collectively facilitate more effective health decisions and navigation of healthcare systems. Consistent with this logic, Mahmoudi and Taheri reported higher health literacy among PhD students compared with Master's students, suggesting that advanced training in research and analytic skills may bolster the capacity to evaluate and apply health information [27]. Our findings align with the view that postgraduate education fosters greater engagement with online resources and peer networks for health information, underscoring the role of digital literacy and social capital in information access. However, it is important to recognize that education likely interacts with other factors (e.g., digital access, health status, information‐seeking habits) to shape health literacy, and the directionality of effects cannot be inferred from cross‐sectional data. Future work should explore potential mediators (e.g., information‐seeking behavior, digital literacy) and moderators (e.g., age, field of study, cultural context) of the education–health–literacy relationship, ideally using longitudinal or experimental designs and harmonized measurement across populations.

The present study found no significant association between health literacy and marital status, suggesting that relationship status did not substantively influence health literacy in this sample. This diverges from some prior work that has linked marital status to higher health literacy particularly among elderly married couples [29] and to associations with marital satisfaction and quality of life in married women [30]. Our null result may reflect sample‐specific factors (e.g., age distribution, cultural context), limited marital‐status categories, measurement issues, or unmeasured confounding variables. It is also possible that the effect of marital status is contingent on other moderators (e.g., social support, living arrangements, or health status) or domain‐specific dimensions of health literacy not captured by our overall measure. We recommend that future research use larger, more diverse samples, harmonized instruments, and consider stratified analyses by age, sex, and context to clarify whether and when marital status influences health literacy. Longitudinal designs and more granular categorizations of marital status could help disentangle potential causal pathways and contextual moderators.

Caution is warranted in interpreting these results, given the study's methodological limitations. The cross‐sectional design prevents causal conclusions, and reliance on self‐reported measures may introduce measurement bias, possibly leading to the overestimation of certain behaviors. Moreover, the sample was largely composed of university staff and individuals with higher educational attainment, potentially reflecting higher baseline health knowledge and reducing the study's generalizability to broader populations. Furthermore, we did not measure or adjust for certain potential confounders, including income, digital access, and health status, which may influence health literacy and literacy‐related constructs. Moreover, the use of self‐reported measures may introduce social desirability or recall bias. Future longitudinal research incorporating socioeconomic, digital access, and health status indicators is needed to validate and extend these findings.

Replicating this study in more diverse samples and across different contexts, employing longitudinal designs, and developing a comprehensive measure of health literacy would advance understanding of the relationships among health literacy, information literacy, physical literacy, and related factors. Such efforts would help determine the generalizability of our findings and illuminate contextual moderators or domain‐specific patterns. Our results can inform the design of health literacy programs and interventions for university employees, with particular attention to strengthening information literacy and physical literacy components as potential leverage points. Policymakers and practitioners can use these insights to create supportive, information‐rich environments that facilitate access to reliable health information, foster critical appraisal skills, and promote health‐promoting behaviors among university staff.

## 5 Conclusion

The study demonstrated that higher information literacy and greater educational attainment are associated with higher health literacy among highly educated university staff in Iran, identifying these factors as key determinants within this context. The robust positive association between health literacy and information literacy suggested that information literacy is a core component of health literacy for this population. Given the specific, context‐rich sample, these findings should be interpreted with caution when generalizing to other settings. Policy and intervention implications should be pursued tentatively and require replication in more diverse populations and contexts. Future work should incorporate broader samples, longitudinal designs, and harmonized measures to examine whether these relationships hold across different educational levels, cultures, and organizational environments, thereby informing context‐appropriate health literacy programs and policies.

## Author Contributions

**Maryam Mohammadi:** helped to edit the manuscript. **Sahar Mohammadnabizadeh:** participated in the writing and design of the study, performed the statistical analysis, and drafted the manuscript. All authors have read and approved the final version of the manuscript. SM had full access to all of the data in this study and takes complete responsibility for the integrity of the data and the accuracy of the data analysis.

## Ethics Statement

The study was approved by the Mashhad University of Medical Sciences ethics board (Number: IR. MUMS. FHMPM. REC.1404.111). In this study, the principles of the Declaration of Helsinki have been followed. A written consent was obtained from all participants after explaining the aim of the study.

## Consent

The authors have nothing to report.

## Funding

The authors have nothing to report.

## Conflicts of Interest

The authors declare no conflicts of interest.

## Transparency Statement

The lead author, Sahar Mohammadnabizadeh, affirms that this manuscript is an honest, accurate, and transparent account of the study being reported; that no important aspects of the study have been omitted; and that any discrepancies from the study as planned (and, if relevant, registered) have been explained.

## Data Availability

The datasets used and/or analyzed during the current study are available from the corresponding author on reasonable request.
